# Association between lifetime smoking and cutaneous squamous cell carcinoma: A 2-sample Mendelian randomization study

**DOI:** 10.1016/j.jdin.2023.11.005

**Published:** 2023-12-03

**Authors:** Truelian Lee, Christopher D. George, Chen Jiang, Maryam M. Asgari, Tamar Nijsten, Luba M. Pardo, Hélène Choquet

**Affiliations:** a Harvard Medical School, Boston, Massachusetts; b Department of Dermatology, Erasmus MC Cancer Institute, University Medical Center Rotterdam, Rotterdam, Netherlands; c Division of Research, Kaiser Permanente Northern California, Oakland, California; d Department of Dermatology, University of Colorado Anschutz Medical Campus, Aurora, Colorado

**Keywords:** cigarette smoking, cutaneous squamous cell carcinoma, genetic epidemiology, Mendelian randomization

## Abstract

**Background/Purpose:**

Cutaneous squamous cell carcinoma (cSCC) is one of the most common malignancies worldwide. While several environmental risk factors for cSCC are well established, there is conflicting evidence on cigarette smoking (and its potential causal effect) and cSCC risk. Furthermore, it is unclear if these potential associations represent causal, modifiable risk factors for cSCC development. This study aims to assess the nature of the associations between cigarette smoking traits (smoking initiation, amount smoked, and lifetime smoking exposure) and cSCC risk using two-sample Mendelian randomization analyses.

**Methods:**

Genetic instruments, based on common genetic variants associated with cigarette smoking traits (*P* < 5 × 10^−8^), were derived from published genome-wide association studies (GWASs). For cSCC, we used GWAS summary statistics from the Kaiser Permanente GERA cohort (7701 cSCC cases and 60,167 controls; all non-Hispanic Whites).

**Results:**

We found modest evidence that genetically determined lifetime smoking was associated with cSCC (inverse-variance weighted method: OR[95% CI] = 1.47[1.09-1.98]; *P* = .012), suggesting it may be a causal risk factor for cSCC. We did not detect any evidence of association between genetically determined smoking initiation or amount smoked and cSCC risk.

**Conclusion:**

Study findings highlight the importance of smoking prevention and may support risk-stratified cSCC screening strategies based on carcinogen exposure and other genetic and clinical information.

**Capsule Summary**

• Considering conflicting evidence regarding the association between cigarette smoking and cSCC, we investigated if there was genetic evidence of a potential causal relationship between cigarette smoking traits and cSCC risk.
• Our findings provide genetic evidence that increased lifetime smoking may be a causal risk factor for cSCC.

## Introduction

Cutaneous squamous cell carcinoma (cSCC) is one of the most common skin cancers worldwide, with an increasing prevalence in recent years.^1^ cSCCs frequently occur in sun-exposed areas such as the head and neck.^2^ According to the Global Burden of Disease (GBD) study, the cutaneous malignancy with the largest worldwide increase in prevalence between 1990 and 2017 was cSCC with a 310% increase.^1^ In a more recent GBD analysis, there was an increase in the global all-age disability-adjusted life years for cSCC in both sexes combined, compared to that in 1990 and 2010.^3^

Besides well-established risk factors for cSCC, including genetic risk factors, such as those affecting pigmentation traits, and environmental factors (e.g., UV radiation exposure and immunosuppression),^4^ cigarette smoking is a potential risk factor for cSCC, as it has been associated with a wide variety of neoplasms.^5^, ^6^, ^7^, ^8^, ^9^ Despite prior observational studies demonstrating a link between smoking and cSCC, the causal association between the 2 remains unclear.^10^, ^11^, ^12^ Additional limitations to prior analyses include selection bias (nonparticipation could be related to smoking amount/status or people who smoke may have higher cumulative UV exposure levels) and difficulty in accurately assessing the amount and duration of smoking (certain studies did not distinguish between cigar and cigarettes, or former, never, and current smokers).^10^, ^11^, ^12^

Compared to observational studies, Mendelian randomization (MR) studies help to assess the causal relation between an exposure and an outcome. In MR, genetic variants are used as instrumental variables (IVs), with the assumption that humans are all randomly assigned genetic variants from their parents.^13^ This is significant because genetic variants, if associated with the exposure and not directly with the outcome and not associated with confounders, can serve as a reliable IV, hence enabling causal inference. Because of the randomization in genetic variants in a population, MR can reduce the effect of confounding, reverse causation, and various biases seen in observational studies.^13^ Although the application of MR rests upon more extensive assumptions than a randomized controlled trial (RCT), MR can be used in cases where randomized controlled trials (RCTs) are not feasible or ethical.^13^^,^^14^

The present study aims to elucidate the causal association of cigarette smoking traits (ie, smoking initiation, amount smoked, and lifetime smoking exposure) with cSCC risk, and circumvent prior limitations, by using a two-sample MR approach. We used 3 separate cigarette smoking traits as the exposures, as those represent different stages of cigarette use: initiation for “smoking initiation,” heaviness for “amount smoked,” and initiation, duration, heaviness, and cessation for “lifetime smoking.” We compare genetic effect estimates for those 3 cigarette smoking traits (exposures) and cSCC risk (outcome) obtained through GWAS summary statistics, especially, from our previous GWAS of cSCC conducted in the Genetic Epidemiology Research on Adult Health and Aging (GERA) cohort.^15^ Through our study, we aim to highlight the potential value of integrating the desire to smoke (determined genetically) in cSCC risk assessment, thereby informing targeted prevention and screening strategies for populations at higher risk of developing the disease.

## Methods

### Study design

Two-sample MR analyses were conducted to investigate separately the association of genetically determined smoking initiation, genetically determined amount smoked, and genetically determined lifetime smoking exposure with the risk of cSCC. For each of the 3 exposures, we used the lead single-nucleotide polymorphisms (SNPs) previously reported as genome-wide significant (*P* < 5.0 × 10^-8^) as a set of genetic instruments. Genetic instruments were then clumped using a window of 10 Mb and maximal linkage disequilibrium of *r*^2^ = 0.001 between instruments to ensure that genetic variants were independent. The different data sets used for this MR study are summarized in Supplementary Table I, available via Mendeley at https://doi.org/10.17632/cnkvc82bgr.1.

### GWAS summary statistics for cSCC

Genetic association data for cSCC risk (outcome) were retrieved from our previous GWAS study conducted in the GERA cohort.^15^ The GERA cohort consists of 110,266 adult members of the Kaiser Permanente Medical Care Plan, Northern California Region (KPNC), an integrated healthcare delivery system, that includes ongoing longitudinal electronic health records (EHRs).^16^^,^^17^ The Institutional Review Board (IRB) of the Kaiser Foundation Research Institute approved all study procedures. Written informed consent was obtained from all participants. In the current study, we retrieved genetic association data from the GWAS of cSCC conducted in 7701 SCC cases and 60,167 controls; all GERA participants of European ancestry.^15^

### Genetic instruments for cigarette smoking traits

Genetic variants as IVs for cigarette smoking initiation (ever having smoked regularly versus never) and amount smoked (number of cigarettes per day) were extracted from the most recent GWAS and Sequencing Consortium of Alcohol and Nicotine use (GSCAN) study.^18^ GWAS summary statistics for smoking initiation and amount smoked analyses,^18^ included 805,431 and 326,497 individuals of European ancestry, respectively, from 42 cohorts (Supplementary Table II, available via Mendeley at https://doi.org/10.17632/cnkvc82bgr.1). Those GWAS summary statistics were publicly accessible at https://conservancy.umn.edu/handle/11299/241912. After clumping, a total of 236 genetic instruments for smoking initiation and 45 for cigarettes per day were used for the MR analyses (Supplementary Tables III-IV, available via Mendeley at xxx). Thus, by using these genetic variants, we adhered to the key assumption that the IVs are robustly associated with the exposure, as those genetic variants were previously reported as genome-wide significant in the large GSCAN study,^18^ making those strong IVs for cigarette smoking initiation and cigarettes per day. Moreover, the genetic scores of the 2 smoking-related traits have been previously reported to be associated with self-reported smoking behaviors in the GERA cohort.^19^

We also used genetic variants as instrumental variables for lifetime smoking (represented by an index which captures smoking status, duration, heaviness, and cessation) from a GWAS conducted in 462,690 UK Biobank (UKB) participants of European ancestry (54% female; mean age (SD) = 56.7 (8.0) years; 54% had never smoked).^20^ The UKB is a longitudinal study following the health of approximately 500,000 participants aged 40-69 years, recruited from across the United Kingdom between 2006 and 2010.^21^ As previously described,^20^ smoking measures available in UKB (ie, smoking status, age at initiation and at cessation, and number of cigarettes smoked per day) were self-reported and collected at initial assessment; smoking measures were then combined into a lifetime smoking index. GWAS summary statistics for this study^20^ were publicly accessible via GWAS Catalog under study accession identifier GCST009096. After clumping, a total of 121 genetic instruments for lifetime smoking were used for the MR analyses (Supplementary Table V, available via Mendeley at https://doi.org/10.17632/cnkvc82bgr.1).

### Genome-wide genetic correlation analyses

We assessed genetic correlations (*r*_*g*_) between the 3 cigarette smoking traits using cross-trait linkage disequilibrium score regression^22^ and using the above-mentioned GWAS summary statistics for smoking initiation and cigarettes per day^18^ and for lifetime smoking.^20^

### Two-sample MR analyses

All analyses were conducted in the R software (V.4.0.1) using the “TwoSampleMR” package.^23^ This package makes causal inference about an exposure on an outcome using GWAS summary statistics, generates LD pruning of exposure SNPs, and harmonizes exposure and outcome data sets. We used the inverse-variance weighted (IVW) method as our primary source of MR estimates. This IVW method essentially translates to a weighted regression of SNP outcome effects on SNP-exposure effects where the intercept is constrained to zero. Moreover, we reported the estimations from MR weighted median, weighted mode, and MR-Egger. Furthermore, leave-one-SNP-out analyses were conducted (Supplementary Tables VI-VIII, available via Mendeley at https://doi.org/10.17632/cnkvc82bgr.1).

### Sensitivity analyses

The potential effect of pleiotropy was evaluated by the regression intercept from the MR-Egger method^24^ and Cochran Q tests were used to evaluate the presence of global heterogeneity amongst the effects of the genetic instruments^25^ (Supplementary Table IX, available via Mendeley at https://doi.org/10.17632/cnkvc82bgr.1). The MR-PRESSO^25^^,^^26^ method was also used to provide an MR estimate which is robust against the presence of heterogeneity among SNP effects and to re-assess the MR estimate after excluding outlier SNPs.

## Results

### Lifetime smoking exposure shares genetic determinants with smoking initiation and cigarettes per day

To quantify genetic overlap between the 3 exposures, genome-wide genetic correlation analyses were performed using cross-trait linkage disequilibrium score regression.^22^ Lifetime smoking exposure was genetically associated with smoking initiation (*r*_*g*_, 0.87; SE, 0.01; *P* = 1.0 × 10^-300^) and cigarettes per day (*r*_*g*_, 0.52; SE, 0.02; *P* = 1.28 × 10^-165^). Consistently, we also found evidence of genetic association between cigarettes per day and cigarette smoking initiation (*r*_*g*_, 0.26; SE, 0.03; *P* = 5.57 × 10^-19^), as previously reported in the GSCAN study.^18^

### Mendelian randomization analyses

**Fig 1:**
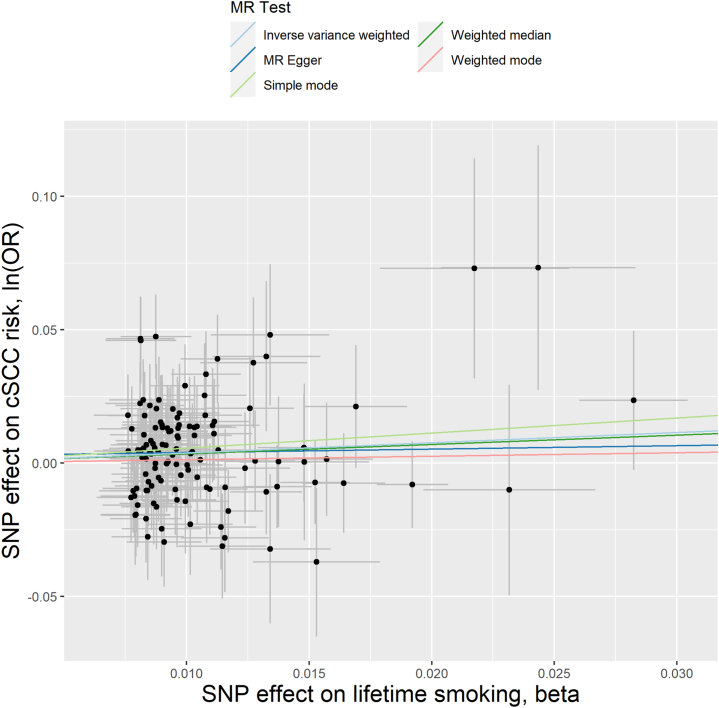
Association of lifetime smoking variants with the risk of cSCC. The x-axis shows 121 genetic instruments for lifetime smoking and their effect size estimates (ORs) with lifetime smoking. The y-axis shows the association of the same variants with cSCC risk. The Mendelian randomization (*MR*) inverse-weighted (*IVW*) regression line is plotted, along with MR Egger, simple mode, and weighted median model. *cSCC*, Cutaneous squamous cell carcinoma; *OR*, odds ratio; *SNP*, single-nucleotide polymorphism.

We conducted two-sample MR analyses to investigate whether cigarette smoking traits causally influenced cSCC risk. No significant association was found between smoking initiation or the number of cigarettes smoked per day and cSCC risk (Table I and Supplementary Figs 1 and 2, available via Mendeley at https://doi.org/10.17632/cnkvc82bgr.1). In contrast, we found evidence for a causal effect of lifetime smoking exposure on cSCC risk, as an increase in lifetime smoking exposure was associated with an increased risk of cSCC (IVW model: odds ratio [OR] per one-unit increase = 1.47; 95% CI, 1.09-1.98; *P* = .012) (Table I and Fig 1).

**Table I:** MR results of the associations of genetically predicted cigarette smoking traits with cSCC risk

Exposure (source)	Outcome	N genetic instruments	MR method	OR (95%CI)	*P*-value	Detected outlier SNP via MR-PRESSO
Cigarette smoking initiation (GSCAN)	cSCC (GERA)	236	IVW	0.99 (0.79-1.25)	.94	
		235	MR-PRESSO model	0.94 (0.86-1.26)	.69	rs7195043
Cigarettes per day (GSCAN)	cSCC (GERA)	45	IVW	0.91 (0.68-1.21)	.50	
		44	MR-PRESSO model	0.96 (0.75-1.23)	.76	rs2016968
Lifetime smoking (UKB)	cSCC (GERA)	121	IVW	1.47 (1.09-1.98)	**.012**	
		121	MR-PRESSO model	1.46 (1.09-1.97)	**.013**	NA

*P*-values reported in this table represent the statistical significance of the association between the exposure of interest (genetically determined smoking trait) and the outcome (cSCC risk). A threshold of *P* < .05 was used to determine significance and bold *P*-values are considered significant.

*CI*, Confidence interval; *cSCC*, cutaneous squamous cell carcinoma; *GERA*, Genetic Epidemiology Research on Adult Health and Aging; *GSCAN*, GWAS and Sequencing Consortium of Alcohol and Nicotine use; *IVW*, inverse-variance weighted model; *MR*, Mendelian randomization; *OR*, odds ratio; *SNP*, single-nucleotide polymorphism; *UKB*, UK Biobank.

### Sensitivity analyses

No evidence of directional or horizontal pleiotropy was observed for all the analyses, as indicated by MR-Egger intercept *P*-values greater than 0.05. Furthermore, using the Cochran Q statistic, no significant heterogeneity was observed among the effects of the genetic instruments for lifetime smoking exposure (Q = 117.58, *P* = .55) (Supplementary Table XI, available via Mendeley at https://doi.org/10.17632/cnkvc82bgr.1). Finally, the MR-PRESSO test detected no outliers for lifetime smoking exposure (Table I).

## Discussion

Using MR on GWAS summary statistics from the Kaiser Permanente GERA cohort (7701 cSCC cases and 60,167 controls, all non-Hispanic whites), we found modest evidence that genetically determined lifetime smoking was significantly associated with cSCC, though there was no evidence of an association between genetically determined smoking initiation and amount smoked (cigarettes per day).

Our results support the findings of previous observational studies, which reported heterogeneous relationships of smoking traits with cSCC risk, depending on the trait tested and study population.^10^, ^11^, ^12^^,^^27^ Although our study used GWAS summary statistics from the largest study of smoking traits published to date (GSCAN consortium),^18^ which provided powerful genetic instruments for MR analyses, we did not observe evidence of causal relationship between smoking initiation or cigarettes per day and cSCC risk. In contrast, we found that genetically determined lifetime smoking was significantly associated with an increased risk of cSCC.

Our MR analyses were performed using valid IVs for causal inference under the 3 assumptions required for MR studies.^28^^,^^29^ The first assumption (ie, the IV should be truly associated with the exposure) was satisfied by the use of genetic variants previously reported as genome-wide significant in large studies,^18^^,^^20^ making those strong genetic instruments for cigarette smoking traits. The second assumption (ie, the IV should not be influenced by any confounders of the exposure-outcome association) was partially satisfied by the fact that we found similar results using the MR-PRESSO method, which is a robust method for sensitivity analysis. The third assumption (ie, the IV should only be related to the outcome of interest through the exposure under study) was satisfied because no evidence of horizontal pleiotropy was detected. Altogether, our findings were unlikely to be affected by the violation of MR assumptions. Nevertheless, recent MR studies^30^^,^^31^ suggested that existing methods for detecting and accounting for horizontal pleiotropy are ineffective under some plausible conditions. Furthermore, genetic instruments for complex behavioral factors such as smoking traits seem to demonstrate horizontal pleiotropy.^32^ Future MR investigations using genetic instruments for smoking traits may include negative control outcomes (ie, an outcome for which it is believed that the exposure cannot be causal) as an approach to avoid the violation of the IV assumptions (such as through pleiotropy).^33^^,^^34^

There are potential limitations to the current study. First, analyzing data sets (for the exposures and outcome) of individuals with the same ancestry (i.e. European ancestry) helped reduce linkage disequilibrium, but our conclusions may have limited applications to individuals of non-European ancestry. Future studies can explore if there is a significant association between smoking traits and cSCC risk in populations of different ancestries. A previous single-center retrospective chart review study suggested a strong association between smoking and age of cSCC diagnosis in non-European populations,^35^ and MR analysis can help further elucidate this possible association. Second, deriving exposures (ie smoking initiation and cigarettes per day) from GWAS summary data combining many heterogeneous cohorts (versus from a unique homogeneous cohort (i.e. UKB for lifetime smoking)) could be considered as a study limitation. Furthermore, cohort characteristics from which exposures were derived would have led to potential selection bias; for instance, lifetime smoking was derived from UKB, with participants being less likely to be a smoker and overall healthier than the general UK population.^36^ Third, in the current study, we did not consider epigenetic changes that could modulate gene expression and distort the effect that MR finds between a genetic variant and an outcome. Thus, future MR studies ideally would incorporate information about epigenetic changes on the genetic variants in the data analysis.^37^^,^^38^ Fourth, we acknowledge the limitations of MR to help determine causal associations given the assumptions that must be made before applying this technique. By assuming that humans are all randomly assigned genetic variants from their parents, we have also assumed that these genes are fully penetrant and not significantly impacted by environmental factors such as UV exposure, diet, or other factors that may influence cSCC risk.^4^^,^^39^ In essence, disease development such as cSCC consists of multifactorial, dynamic, nonlinear biological processes, and MR is limited in accounting for that complexity. Future studies can expand on this research by analyzing the interplay between genetic predispositions for smoking, UV exposure, diet, and other behaviors on cSCC risk.

Although no causal associations of smoking initiation and smoking amount with SCC risk were detected, we observed support for a causal association of lifetime smoking with SCC risk. Lifetime smoking exposure is based on an index that incorporates total duration of smoking, time since smoking cessation, average number of cigarettes smoked per day, and selected covariable interactions to form an aggregate measurement.^40^ For this reason, we feel that lifetime smoking might be a more comprehensive exposure for smoking behavior, compared to smoking initiation and amount smoked per day. Previous MR studies have reported causal associations between lifetime smoking exposure and other cancers, including breast cancer and colorectal cancer, but a lack of association with other cigarette smoking traits.^20^^,^^21^ Thus, future MR studies that aim to investigate the causal relationship between cigarette smoking and outcomes should test for different smoking traits, including lifetime smoking index, as those represent different stages of cigarette use.

In this study analyzing GWAS summary statistics of different cohorts through two-sample MR, we found support for a causal relationship between long-term smoking exposure and cSCC risk. These results support more targeted screening strategies of populations based on factors such as carcinogen exposure and genetic and clinical information. Given that lifetime smoking exposure is an aggregate measurement incorporating total duration of smoking, time since smoking cessation, average number of cigarettes smoked per day, and selected covariable interactions, our results also support an overall reduction of smoking exposure. This conclusion may have broader implications in highlighting the importance of smoking cessation initiatives in decreasing smoking exposure. Causal relationships between smoking traits and a wide range of cancers, including cancers of the lung, head and neck, esophagus, pancreas, bladder, kidney, cervix, and ovaries, and myeloid leukemia, have been reported,^22^ and our results can potentially provide additional evidence for skin cancer. In conclusion, this study shows evidence for a causal relationship between long-term smoking exposure and cSCC risk, underscoring the importance of smoking prevention campaigns and more targeted screening strategies.

## Conflicts of interest

None disclosed.
